# Response of a Human Lens Epithelial Cell Line to Hyperglycemic and Oxidative Stress: The Role of Aldose Reductase

**DOI:** 10.3390/antiox12040829

**Published:** 2023-03-28

**Authors:** Gemma Sardelli, Viola Scali, Giovanni Signore, Francesco Balestri, Mario Cappiello, Umberto Mura, Antonella Del Corso, Roberta Moschini

**Affiliations:** 1 Biochemistry Unit, Department of Biology, University of Pisa, 56123 Pisa, Italy; 2 Interdepartmental Research Center Nutrafood “Nutraceuticals and Food for Health”, University of Pisa, 56124 Pisa, Italy

**Keywords:** hyperglycemia, oxidative stress, aldose reductase, 4-hydroxy-2-nonenal

## Abstract

A common feature of different types of diabetes is the high blood glucose levels, which are known to induce a series of metabolic alterations, leading to damaging events in different tissues. Among these alterations, both increased polyol pathway flux and oxidative stress are considered to play relevant roles in the response of different cells. In this work, the effect on a human lens epithelial cell line of stress conditions, consisting of exposure to either high glucose levels or to the lipid peroxidation product 4-hydroxy-2-nonenal, is reported. The occurrence of osmotic imbalance, alterations of glutathione levels, and expression of inflammatory markers was monitored. A common feature of the two stress conditions was the expression of COX-2, which, only in the case of hyperglycemic stress, occurred through NF-κB activation. In our cell model, aldose reductase activity, which is confirmed as the only activity responsible for the osmotic imbalance occurring in hyperglycemic conditions, seemed to have no role in controlling the onset of the inflammatory phenomena. However, it played a relevant role in cellular detoxification against lipid peroxidation products. These results, in confirming the multifactorial nature of the inflammatory phenomena, highlight the dual role of aldose reductase as having both damaging but also protecting activity, depending on stress conditions.

## 1. Introduction

Diabetes mellitus (DM) is a widespread chronic disease, affecting hundreds of millions of people around the world. As reported by the World Health Organisaton (WHO), in 2019, 1.5 million deaths could be directly ascribed to DM; moreover, 48% of these deaths occurred in people below the age of 70 [1]. Irrespective of its classification as type 1 or type 2, DM is characterized by an abnormally high concentration of glucose in the blood, defined as “hyperglycemia”, which is responsible for severe damaging metabolic alterations occurring at the level of different organs. Among those alterations recognized as usually occurring in diabetic subjects are an increase in advanced glycation end-products, the activation of protein kinase C, and of both the hexosamine and the polyol pathways [2]. The increased flux through the polyol pathway has also received special attention due to its well-established involvement in the onset of the so-called diabetic complications, which include neuropathy, nephropathy, cataracts, and retinopathy [3,4,5]. The polyol pathway is a two-step metabolic route, allowing D-glucose to be firstly reduced to sorbitol, due to the catalytic action of the NADPH-dependent aldose reductase (AKR1B1), and then subsequently oxidized to D-fructose, due to the catalytic action of the NAD^+^-dependent sorbitol dehydrogenase (SDH). In hyperglycemic conditions, it is estimated that the flux through the polyol pathway significantly increases, so that almost 30% of metabolized glucose enters this pathway [6], which in normoglycemic conditions receives less than 3% of metabolized glucose [7]. This leads to sorbitol accumulation, and the resulting osmotic imbalance is considered a relevant event in the series of metabolic alterations leading to the onset of diabetic complications [8,9]. Indeed, inhibition of AKR1B1, or its genetic ablation in animal models, resulted in an overall protection against diabetic complications, both in terms of delay of their onset and of extent of their damage [10,11,12]. However, AKR1B1, besides its damaging role linked to its involvement in the polyol pathway, is strongly involved in the removal of toxic aldehydes, such as those generated during lipid peroxidation [13,14]. This dual role poses the question of the overall advantage deriving from AKR1B1 inhibition, and has led to the proposal of targeting AKR1B1 through a “differential inhibition approach”, aimed at blocking the enzyme’s action on glucose, without affecting the action on toxic aldehydes [15,16].

As a general consequence of hyperglycemic conditions, the occurrence of both oxidative stress [17,18,19,20] and, through the activation of different transcription factors, inflammation [20,21,22,23,24,25], has been reported. Concerning oxidative stress, the lipid peroxidation product 4-hydroxy-2-nonenal (HNE), deserves special attention, not only for being a relevant general product in oxidative stress conditions [26], but also for its role in favoring insulin resistance [27]. Moreover, HNE has been recently proposed as a relevant factor, originating in DM, possibly representing a link between this disease and cancer [28]. This aldehydic compound can be metabolized either through its conjugation with glutathione via Michael addition, which occurs both spontaneously and upon the action of different glutathione S-transferases [29,30], to generate 3-glutathionyl-4-hydroxynonal (GSHNE), or upon red/ox transformations (occurring both on HNE and on GSHNE) catalyzed by oxidoreductases, whose abundance and specificity are strictly tissue-dependent [31,32,33,34,35]. In particular, concerning GSHNE, besides AKR1B1, which has been demonstrated to be able to participate not only to HNE but also in the adduct reduction [13], carbonyl reductase 1 (CBR1) has been more recently indicated as being effective in catalyzing GSHNE transformation [36,37,38].

Concerning inflammation, nuclear factor kappa-light-chain-enhancer of activated B cells (NF-κB), is a transcription factor widely reported to be causative of the transcription of genes involved in the inflammatory response [39]. In particular, NF-κB activation has been observed in several cell systems exposed to a hyperglycemic stimulus [40]. Two different hypotheses have been raised to link high glucose stress (and more generally oxidative stress) and the NF-κB dependent inflammation. The first one dates back to the first decade of this millennium and is based on the cytotoxic effect exerted by 3-glutathionyl-dihydroxynonane (GSDHN), i.e., the reduced form of GSHNE, through NF-κB-dependent signaling [41,42]. The cytotoxicity was suggested to occur through the stimulation of the phospholipase C/protein kinase C pathway [43]. In this case, the in vitro ability of AKR1B1 to reduce the GSHNE adduct [13], was the rationale for the impairment of NF-κB activation observed in some cell models upon AKR1B1 inhibition [44,45,46,47]. Several aspects of this proposal still remain to be fully clarified, such as a final assessment of the extent of involvement, in different cell systems, of CBR1, able (as stated above) to intervene in the reduction of GSHNE. Moreover, a relevant contribution of chirality has been assessed at different steps of HNE metabolism [48,49,50], and this may also affect GSHNE metabolism. Indeed, a further aspect still to be clarified, is the possible different effects of different GSDHN diastereoisomers, originating in GSHE metabolism, in mediating the inflammatory response. In fact, the supposed effect of GSDHN in affecting phospholipase C, thus activating the signalling cascade leading to NF-κB activation, has never been studied in terms of the chiral requirements of GSDHN. Concerning the other hypothesis raised to connect high glucose stress and the NF-κB dependent inflammation, it is related to the fact that the expression of several inflammatory signaling genes results from the stimulation by hyperacetylation of NF-κB [51]. Recently, an alteration of sirtuin-mediated NF-κB acetylation, linked to a disequilibrium of the NAD^+^/NADH ratio, due to the increased polyol pathway flux, has been hypothesized to connect hyperglycemia and NF-κB expression [52].

In this work the effects of both hyperglycemic stress and HNE-induced oxidative stress were evaluated on a human lens epithelial cell line, and the role of AKR1B1 in the onset of osmotic imbalance and inflammation was considered. Our results confirm the central role of AKR1B1 as causative of sorbitol accumulation, consequent on the increased flux through the polyol pathway. At the same time, the enzyme is relevant in helping normal cell function, due to its action in the detoxification of lipid peroxidation products. On the other hand, our data raise several doubts concerning an unequivocal role of AKR1B1 in the onset of inflammatory phenomena.

## 2. Materials and Methods

### 2.1. Materials

Cell culture media, fetal bovine serum (FBS), penicillin/streptomycin solution, gentamicin, and glutamine were purchased from Euroclone (Pero, Italy). HNE was synthesized as previously described [48]. GSHNE was synthesized as described [53]. NADP^+^, NAD^+^, NADPH, and NADH were from Carbosynth (Compton, UK). D-glucose, reduced glutathione (GSH), glutathione disulfide (GSSG), bovine serum albumin, D,L-glyceraldehyde (GAL), D,L-dithiothreitol (DTT), sorbinil, 3-(4,5-dimethylthiazol-2-yl)-2,5-diphenyltetrazolium bromide (MTT), phenylmethylsulfonide fluoride (PMSF), phosphate buffered solution (PBS), and hygromycin were from Merck Life Science (Milan, Italy). All inorganic chemicals were of reagent grade, from VWR (Poole, Dorset, UK).

### 2.2. Cell Cultures and Transfection

The human lens epithelial line B3 (ATCC CRL-11421) cells were obtained from ATCC (Rockville, MD, USA), and stably transfected with a pGL4.32[luc2P/NF-κB-RE/Hygro] plasmid (HLE). The plasmid contained the hygromycin and ampicillin resistance genes and five copies of the NF-κB response element (NF-κB-RE), which, consequently to NF-κB binding, regulates the transcription of the *luc2P* reporter gene, encoding the luciferase of *Photinus pyralis.* HLE cells were routinely cultured at 37 °C in a humidified atmosphere, in the presence of 5% CO_2_, in an Eagle’s modified minimum essential medium (MEM) containing 5.5 mM D-glucose, supplemented with 20% (*v/v*) FBS, 50 mU/mL penicillin/streptomycin, 2 mM glutamine and 100 μg/mL hygromycin. Cells were grown in the indicated medium until they were 70% confluent. Before experiments, cells were growth arrested for 24 h by incubation in MEM containing 0.5% FBS and 50 μg/mL gentamicin, 2 mM glutamine, and 100 μg/mL hygromycin. Cells at passages 15–20 (30–40 residual doublings), plated at a density of 20,000 cells/cm^2^, were used for experiments.

### 2.3. Measurement of Cell Vitality

Cell vitality was measured either through the MTT assay [54] or through the crystal violet assay [55]. Briefly, in the case of the MTT assay, HLE cells were incubated with 0.5 mg/mL MTT solution for 30 min at 37 °C, in a humidified 5% CO_2_ atmosphere. Then, the formazane crystals were dissolved by addition of 0.04 N HCl in isopropanol, and the absorbance at the wavelength of 563 nm was measured through a microplate reader. Concerning the crystal violet assay, HLE cells were rinsed twice with PBS. Then, a 200 μL aliquot of 0.5% (*w*/*v*) crystal violet staining solution in methanol was added and the plate was incubated for 15 min at room temperature under gentle agitation. The plate was washed with tap water four times and subsequently air-dried at room temperature for 24 h. After adding 600 μL of 10% (*v/v*) acetic acid to each well, the plate was incubated under agitation for 10 min at room temperature, in order to solubilize the crystal violet dye, and the absorbance at 596 nm was measured through a microplate reader.

### 2.4. Quantification of the Firefly Luciferase Expression

The activation of NF-κB was evaluated by measuring the expression of the Firefly luciferase reporter gene, which resulted in a luminescent signal, detectable through a luminometer. Before analysis, the incubation medium was removed, cells were rinsed with PBS, and incubated with Passive Lysis Buffer (Promega, Madison, WI, USA), according to the protocol provided by manufacturer (Promega, Technical Bulletin 281 Revised 8/15). Then, a 100 μL aliquot of Luciferase Assay System (Promega) was added to 20 μL of the cell lysates, at 37 °C, for assaying luciferase activity. Moles of luciferase were calculated referring to a standard curve, obtained by measuring the luminescence of different amounts of a commercially available Firefly luciferase (QuantiLuM, Promega), in the mole range between 10^−^^15^ and 10^−^^19^. The measured moles of luciferase were normalized to the protein content of each sample.

### 2.5. Sorbitol Dehydrogenase Assay and Purification

The activity of sorbitol dehydrogenase (SDH) was measured at 37 °C, following the conversion of D-sorbitol to D-fructose at 340 nm. The reaction mixture (0.7 mL final volume) contained 0.24 mM NAD^+^ and 0.1 M D-sorbitol, in 100 mM Tris-HCl buffer pH 8. The reaction was initiated by addition of the substrate. One unit (U) of enzyme activity is defined as the amount of SDH that catalyzes the reduction of 1 μmol/min of NAD^+^ in the above conditions. The human recombinant SDH, was purified to electrophoretic homogeneity according to Marini et al. [56], with minor modifications, as detailed in Supplementary Materials (Figures S1–S3). The specific activity of the purified enzyme was 17.2 ± 2 U/mg.

### 2.6. Sorbitol Determination

HLE cells, after removal of the incubation medium and rinsing with PBS supplemented with 1 mM PMSF, were collected with a scraper. Cellular lysis was performed by three cycles of freezing and thawing, followed by centrifugation at 4 °C for 15 min at 14,000× *g*. Samples were supplemented with 1 M perchloric acid and then centrifuged at 4 °C for 5 min, 14,000× *g*. The supernatant was brought to pH 7.0 by addition of KOH, and again centrifuged at 4 °C for 15 min at 14,000× *g*. For the determination of sorbitol content, proper aliquots of the supernatant were added to a reaction mixture (final volume 200 μL) containing 0.24 mM NAD^+^ and 150 mU of SDH in 100 mM Tris-HCI buffer, pH 8. The reaction was initiated by the addition of NAD^+^. The fluorescence emission signal at 460 nm, after excitation at 355 nm for a total time of 10 min at 37 °C, was recorded. The moles of D-sorbitol were calculated referring to a calibration curve obtained using known D-sorbitol concentrations in the range 5–40 µM and were normalized to protein content.

### 2.7. Western Blotting

HLE cells, after removal of the incubation medium and rinsing with PBS supplemented with 10 mM NaF, 10 mM Na_4_O_7_P_2_, 2 mM Na_3_VO_4_, 33 mM β-glycerophosphate, and 1 mM PMSF, were incubated at room temperature for 5 min in Lysis Buffer (Cell Signaling, Danvers, MA, USA), according to the protocol provided by the manufacturer. The obtained cell lysates were then transferred in Eppendorf tubes, kept on ice for 10 min, and then centrifuged at 4 °C for 10 min at 14,000× *g*. Protein denaturation was achieved by dilution of samples in loading buffer (250 mM Tris-HCl, pH 6.8 with 10% (*w*/*v*) SDS, 30% (*v*/*v*) glycerol, 0.05% (*w*/*v*) bromophenol blue salt) and 0.7 M β-mercaptoethanol, followed by incubation at 70 °C for 10 min. Aliquots (25 μg) were loaded on 12% TGX Stain-Free™ FastCast™ Acrylamide Gels (Bio-Rad Laboratories, Hercules, CA, USA) and the electrophoretic separation was performed at a constant voltage of 200 V. Proteins were then transferred to a 0.2 μm PVDF membrane, using precast Trans-Blot Turbo Transfer Pack Midi kit (Bio-Rad), by applying a constant current of 1.3 A with a maximum voltage of 25 V for 6 min, using a Trans-Blot Turbo Transfer System (Bio-Rad). The membrane was incubated for 5 min at room temperature once in 50 mM pH 7.5 Tris-HCl supplemented with 150 mM NaCl (TBS), and three times in TBS supplemented with 0.1% (*v*/*v*) Tween^®^ 20 (TBST). Then, an incubation in the presence of 5% (*w*/*v*) milk powder in TBST, for 1 h at room temperature, was performed. Subsequently, the membrane was incubated for 5 min with TBST three times, and then overnight at 4 °C with the anti-COX-2 antibody (Cell Signaling, 1:1000 in 5% (*w*/*v*) milk powder in TBST). After three incubations at room temperature in TBST for 5 min, the membrane was incubated for 1 h with an HRP-linked anti-rabbit antibody (Cell Signaling, 1:1000 in 5% (*w*/*v*) milk powder in TBST). Finally, the membrane was subjected to three and two incubations of 5 min each at room temperature in TBST and TBS, respectively. Immobilon^TM^ Western Chemiluminescent HRP substrate (Merck) was used for detecting immunoreactive bands through chemiluminescence and a Chemi-Doc Image System device (Bio-Rad). The quantification of the immunoreactive bands was performed by means of the Bio-Rad ImageLab software, using the Stain free (Bio-Rad) technology as loading control [57].

### 2.8. Glutathione Determination

The determination of glutathione was performed by capillary electrophoresis (HPCE), essentially as described in [58], on a PACE/MDQ Beckman instrument, equipped with a 50 cm length and 74 μm inner diameter capillary, using a 100 mM Tris borate pH 8.5 as separation buffer. The anode pressure injection was 0.5 psi for 20 s. Samples were run at 25 °C, using a constant current voltage of 30 kV, for 15 min. Detection was carried out at 214 nm and the concentration of the analytes was determined on the basis of peak areas, through proper calibration curves. HLE cells, after medium removal, were rinsed twice with PBS containing 1 mM PMSF, harvested with a scraper, and immediately stored at −80 °C until use. Crude extracts were obtained through three cycles of freezing and thawing, followed by a 10,000× *g* centrifugation at 4 °C for 30 min. The supernatant is referred to as the crude extract, on which GSH content was evaluated after acidification by the addition of 0.03 N HCl. The solution was then subjected to ultrafiltration, using Amicon Microcon 3 kDa devices (Merck). To measure the concentration of total glutathione, crude extracts were incubated for 2 h at room temperature with 5 mM DTT, before acidification and subsequent ultrafiltration. Samples were analyzed at least in triplicate.

### 2.9. Other Methods

Protein concentration was determined according to Bradford [59], using a Bio-Rad protein assay kit, with a calibration curve obtained using bovine serum albumin as the standard. HNE quantification was performed by a colorimetric method [60], measuring the absorbance at 586 nm of the chromophore generated by the reaction of the aldehyde with 1-methyl-2-phenylindole under acidic conditions. Statistical analysis was performed using GraphPad Prism version 7.04 (GraphPad Software, San Diego, CA, USA), through Student’s *t*-test or one-way ANOVA with a Tukey post hoc test, as specified in the figure legends. Differences with *p* ≤ 0.05 were considered to be statistically significant.

## 3. Results and Discussion

### 3.1. Exposure of HLE Cells to Hyperglycemic Stress

**Figure 1:**
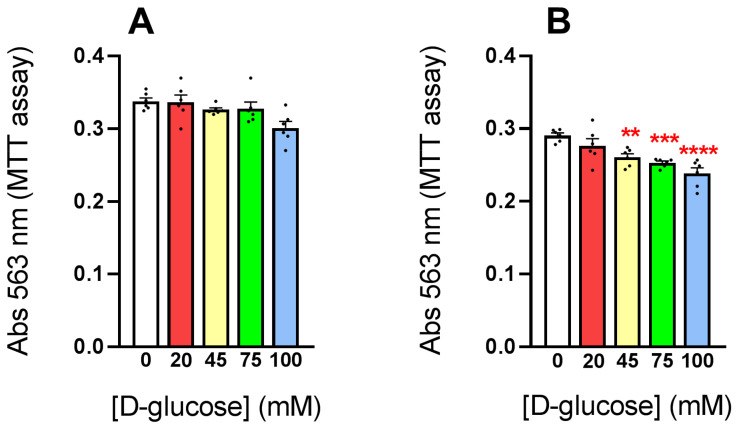
Effect of high glucose exposure on HLE cells’ viability. HLE cells were grown as described in Section 2.2 and maintained for 24 h (**A**) and 48 h (**B**) in MEM supplemented with the indicated D-glucose concentrations. Cell viability was evaluated as described in Section 2.3. All values are reported as the mean ± SEM of six independent measurements. Statistical analysis was performed using one-way ANOVA with Tukey post hoc test. Significance was evaluated with respect to cells incubated in MEM alone (**: *p* < 0.01; ***: *p* < 0.001; ****: *p* < 0.0001).

In order to evaluate the effect on HLE cells of the exposure to high glucose concentrations (in the range 20–100 mM), cell viability was preliminarily monitored after 24 and 48 h of treatment. The results shown in Figure 1A, indicate that no significant effects were observed, at least for concentrations up to 100 mM, after 24 h of treatment. For a 48 h incubation, D-glucose concentrations higher than 20 mM had a slight effect on cell viability. In fact, significant, even though modest, reductions in cell viability, of 10, 12, and 17%, were observed upon incubation of HLE cells in a MEM supplemented with 45, 75, and 100 mM D-glucose, respectively (Figure 1B).

**Figure 2:**
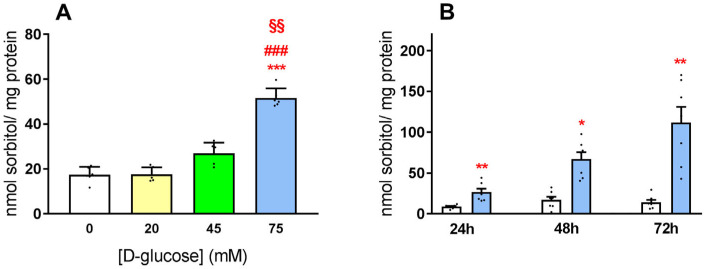
Intracellular sorbitol accumulation in HLE cells exposed to high glucose conditions. Intracellular sorbitol content was fluorimetrically measured, as described in Section 2.6. (**A**) HLE cells were grown for 48 h in MEM supplemented with the indicated D-glucose concentrations. Values are reported as the mean ± SEM of six independent measurements. Statistical analysis was performed using one-way ANOVA with Tukey post hoc test. Significance of the 75 mM D-glucose treatment was evaluated with respect to the following conditions: no treatment: *; 20 mM D-glucose treatment: #; 45 mM D-glucose treatment: ^§.^ (^§§^: *p* < 0.01 value; ***, ^###^: *p* < 0.001). (**B**) Cells were incubated for the indicated times in MEM alone (white bars) or supplemented with 75 mM D-glucose (blue). Values are reported as the mean ± SEM of seven independent measurements. Statistical analysis was performed using Student’s *t*-test. Significance was evaluated with respect to untreated cells incubated for the same time (*: *p*< 0.05; **: *p* < 0.01).

The effect of high glucose exposure was evaluated in terms of increased flux through the polyol pathway, upon the determination of intracellular sorbitol content, using an enzymatic fluorimetric method, based on the ability of SDH to transform sorbitol with a parallel reduction of NAD^+^. The results in Figure 2A, show that intracellular sorbitol content progressively increased with the increase in D-glucose concentration. A significant increase was measured in HLE cells after 48 h exposure to 75 mM D-glucose. Indeed, using this concentration of D-glucose, a significant, time-dependent accumulation of sorbitol was observed for incubation times ranging from 24 to 72 h (Figure 2B). At the longest incubation time, the intracellular sorbitol content increased approximately 9-fold relative to the basal value (i.e., that measured in the presence of the minimum medium containing 5.5 mM D-glucose).

**Figure 3:**
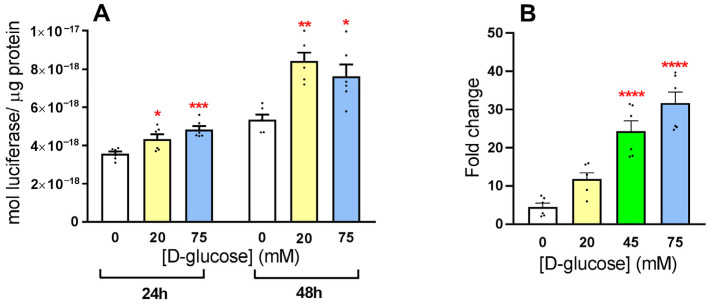
High glucose treatment induced an inflammatory response in HLE cells. HLE cells were grown in MEM (see Section 2.2) supplemented with the indicated D-glucose concentrations. Values are reported as the mean ± SEM of six independent measurements. Statistical analysis was performed using one-way ANOVA with Tukey post hoc test. Significance was evaluated with respect to untreated cells (*: *p* < 0.05; **: *p* < 0.01; ***: *p* < 0.001; ****: *p* < 0.0001). (**A**) The expression of NF-κB was measured at the indicated times, through luminometric detection (see Section 2.4) and reported as mol of luciferase/μg of protein. (**B**) The expression of COX-2 was measured through Western blot (see Section 2.7), 48 h after the addition of glucose. For each condition, data are reported as fold change with respect to COX-2 expression, measured at time zero of incubation.

As detailed in the Introduction, high glucose exposure has been widely considered a stimulus able to induce an inflammatory response, which, depending on the cell system adopted, has been linked to the expression of several different markers. Here, we selected the activation of NF-κB, which in our cell system was linked to Firefly luciferase expression (see Section 2.2), and the expression of COX-2, to evaluate the occurrence of inflammatory phenomena. Indeed, a marked increase in the activation of NF-κB was observed upon 24 and 48 h exposure of HLE cells, both to 20 and 75 mM D-glucose (Figure 3A); NF-κB activation was also significant after 16 h of exposure to 20 mM D-glucose. In addition, COX-2 expression was significantly enhanced upon exposure to high glucose conditions (Figure 3B); the expression of the inflammatory marker in the presence of the highest D-glucose concentration tested, was approximately six times the basal value. For both inflammatory markers, our results indicated that inflammation is induced by a stimulus whose entity did not appear sufficient to determine a significant sorbitol accumulation. In fact, a significant increase in NF-κB and COX-2 expression was observed upon treatment with D-glucose concentrations as low as 20 and 45 mM, respectively.

**Figure 4:**
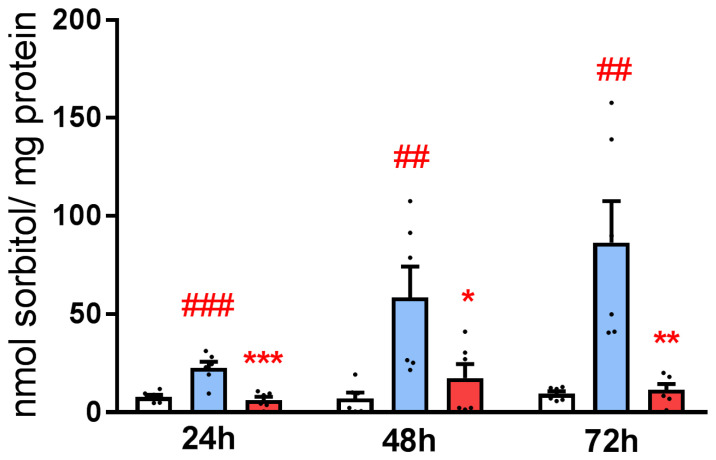
Effect of AKR1B1 inhibition on intracellular sorbitol accumulation. Intracellular sorbitol content was measured through fluorimetric detection (see Section 2.6) in HLE cells grown as described in Section 2.2, and exposed to different treatments for the indicated times; white bars: HLE cells incubated in MEM in the presence of 0.05% (v:v) DMSO; blue bars: cells incubated in MEM supplemented with 0.05% DMSO and 75 mM D-glucose; red bars: cells incubated in MEM supplemented with 0.05% DMSO, 75 mM D-glucose, and 100 µM sorbinil. All values are reported as the mean ± SEM of six independent measurements. Statistical analysis was performed using one-way ANOVA with Tukey post hoc test. Significance was evaluated with respect to cells grown in normal conditions (##: *p* < 0.01 value; ###: *p* < 0.001) and with respect to cells exposed to 75 mM D-glucose (*: *p* < 0.05; **: *p* < 0.01; ***: *p* < 0.001).

The relevance of AKR1B1 activity in determining the increased polyol pathway flux, is clearly shown in Figure 4, in which the time-dependent sorbitol accumulation was monitored both in the absence and in the presence of the AKR1B1 inhibitor sorbinil. These results, confirming the occurrence of the intracellular sorbitol accumulation reported in Figure 2, show that 100 µM sorbinil, added at the beginning of the high glucose treatment, almost completely impaired sorbitol accumulation for all incubation times. A comparable effect was observed in the presence of 50 µM of the AKR1B1 inhibitor. No effect of sorbinil, up to 100 µM, was observed on cell viability (Supplementary Materials, Figure S4). Moreover, no effect of DMSO, used for sorbinil solubilization, was observed on sorbitol accumulation, for solvent concentrations up to 0.1% (v:v), indicating that, in the adopted conditions, the solvent, which has been reported to affect AKR1B1 activity [61], did not significantly interfere with the polyol pathway flux. Lower sorbinil concentrations were able to significantly reduce the polyol accumulation. In fact, 10 µM sorbinil reduced by approximately 50% the sorbitol accumulation observed after 24 h of high glucose exposure (Supplementary Materials, Figure S5). This effect was significantly potentiated upon a 24 h preincubation of 10 µM sorbinil before high glucose treatment. In fact, in the latter condition, basal sorbitol levels were restored (Supplementary Materials, Figure S5). Thus, these results clearly confirmed AKR1B1 as the only entity responsible for the sorbitol accumulation subsequent to the exposure to hyperglycemic stress.

**Figure 5:**
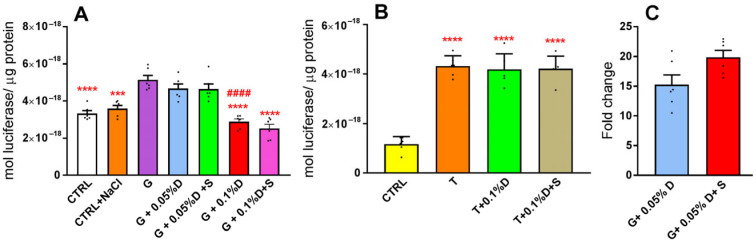
Effect of AKR1B1 inhibition on inflammatory response. (**A**) The expression of NF-κB, reported as mol of luciferase/mg of protein, was measured through luminometric detection (see Section 2.4) after 24 h. CTRL and CTRL + NaCl refer to cells grown in MEM alone or supplemented with 14.4 mM NaCl, respectively. Different letters refer to the following addition to MEM: G, 75 mM D-glucose; D, DMSO; S, 50 μM sorbinil. Values are reported as the mean ± SEM of six independent measurements. Statistical analysis was performed using one-way ANOVA with Tukey post hoc test. Significance was evaluated with respect to G (***: *p* < 0.001; ****: *p* < 0.0001) or with respect to G + 0.05%D (^####^: *p* < 0.0001). (**B**) The expression of NF-κB, reported as mol of luciferase/mg of protein, was measured through luminometric detection (see Section 2.4) after 24 h. CTRL refers to cells grown in MEM containing 175 ng/mL human serum albumin. Different letters refer to the following addition to CTRL: T, 0.2 nM TNF-α; D, DMSO; S: 100 μM sorbinil. Values are reported as the mean ± SEM of six independent measurements. Statistical analysis was performed using one-way ANOVA with Tukey post hoc test. Significance was evaluated with respect to CTRL (****: *p* < 0.0001). (**C**) The expression of COX-2 was measured through Western blot (see Section 2.7) 48 h after the addition of glucose. G: 75 mM D-glucose; D: DMSO; S: 100 μM sorbinil. For each condition, data are reported as fold change with respect to expression values measured at time zero of incubation. Values are reported as the mean ± SEM of four independent measurements. Statistical analysis was performed using Student’s *t*-test.

Despite AKR1B1 inhibition having been shown to reduce inflammation in several cell systems [44,45,46,47], in HLE cells, neither 50 μM (Figure 5A) nor 100 μM sorbinil (Supplementary Materials, Figure S6) had any effect on the NF-κB activation induced by exposure to 75 mM D-glucose. Similarly, no effect was observed when HLE cells were subjected to a 24 h preincubation with 100 µM sorbinil before the exposure to high glucose conditions (Supplementary Materials, Figure S6). The addition to the culture medium of untreated cells of 14.4 mM NaCl, a concentration sufficient to reach the osmolarity of a 28 mM D-glucose solution, did not induce any significant increase in NF-κB activation (Figure 5A).

In addition, COX-2 expression was unaffected by sorbinil treatment, as observed in Figure 5C, in which 75 mM D-glucose was again used to induce the inflammatory response. Moreover, no effect of either 100 μM sorbinil or 25 μM epalrestat, was observed on COX-2 expression when HLE cells were exposed to 20 mM D-glucose. Thus, the inefficacy of AKR1B1 inhibition in impairing the inflammatory response was confirmed also when a milder stimulus was adopted. Figure 5A also reports an effect of DMSO worth noting. In fact, 0.1% DMSO significantly reduced NF-κB activation, restoring basal values of the transcription factor and apparently counteracting the onset of the high-glucose-induced inflammation. This evidence, besides confirming reported observations on the anti-inflammatory action of DMSO [62,63], underlines the importance of the use of DMSO-treated cells as control. In fact, a significant reduction in NF-κB activation would be observed upon comparing the action of sorbinil (which is solubilized in DMSO), with cells cultured in the absence of DMSO. However, making a proper comparison, no effect of sorbinil on NF-κB activation was observed, irrespective of DMSO concentration. The apparent “anti-inflammatory” effect of DMSO was limited to NF-κB activation, since COX-2 expression was not affected by the solvent.

Figure 5B reports, as a control, the response of HLE cells to TNF-α exposure, a condition known to elicit an inflammatory response [64]. Indeed, the TNF-α treatment was sufficient to markedly increase NF-κB activation. In addition, in this condition, as observed when high glucose was used as an inflammatory stimulus, the response to TNF-α was not affected by sorbinil. No effect of 0.1% DMSO was observed on the TNF-α induced inflammatory response. Thus, despite the clearly assessed fundamental role of AKR1B1 in determining the first metabolic alteration subsequent to the high glucose exposure (i.e., sorbitol accumulation, Figure 4), the complete inhibition of the enzyme was not sufficient, in our cellular model, to impair the onset of an inflammatory response. The observed inability to affect the onset of inflammation may be a peculiar response of our cell model with respect to other cells systems, in which AKR1B1 inhibition has been connected with an at least partial impairment of the NF-κB-mediated inflammatory response [44,45,46,47]. The high complexity of the cell inflammatory response makes a direct comparison with other cell systems very difficult, due to differences in the experimental conditions adopted, such as, for example, the overall type of treatment or the different susceptibility of cells to glucose treatment. Thus, for example the stress conditions adopted here, did not appear sufficient to massively interfere with cell viability (Figure 1), differently from what has been reported using even lower D-glucose concentrations [44]. This is an aspect which suggests how different the situation may be, even for cells exposed to similar stresses.

Moreover, the evaluation in HLE cell extracts, of the levels of oxidoreductases involved in HNE/GSHNE metabolism [65], indicated the presence not only of AKR1B1, but also of CBR1, as entities able to act on GSHNE. In fact, as stated in the Introduction, CBR1 is able to participate in the reduction not only of GSHNE, but, more generally, of glutathionylated aldehydes [36,37]. It is worth noting, that 50 μM sorbinil did not affect the activity of the human recombinant purified CBR1 at all; moreover, a significant residual reductase activity on GSHNE remained functioning when HLE cell extracts were incubated in the presence of sorbinil. Thus, considering the proposed link between NF-κB and GSDHN [41,42], the observed lack of action of sorbinil in our cell model could be ascribed to the contribution of an active CBR1. Moreover, AKR1B1 displays a marked stereoselectivity toward different GSHNE diastereoisomers, being able to preferentially convert the specific diastereoisomer 3S,4R-GSHNE [48]. At present, no evidence is available on the possible different ability of different GSHNE diastereoisomers in mediating the NF-κB-dependent signalling. Thus, differences in AKR1B1/CBR1 levels among different cell lines may affect, not only the extent of the overall GSHNE metabolism, but eventually, also, the efficacy in inducing an inflammatory response.

### 3.2. Exposure of HLE to Oxidative Stress

As underlined in the Introduction, oxidative stress is universally recognized as a relevant component of high-glucose-induced stress. In order to evaluate what occurs to HLE cells when exposed to an oxidative stimulus in the absence of the high glucose component, we treated HLE cells with different HNE concentrations.

**Figure 6:**
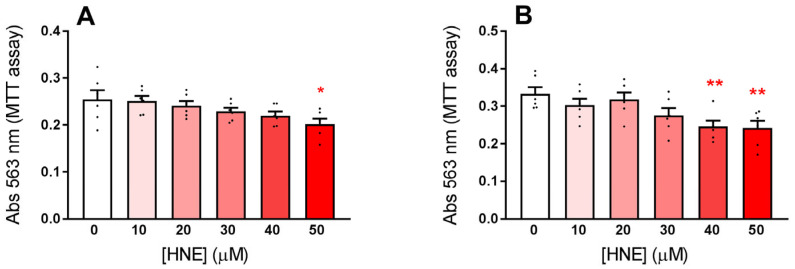
Effect of HNE on HLE cell viability. HLE cells were grown as described in Section 2.2 and maintained in MEM in the presence of the indicated HNE concentrations for 6 h (**A**) and 24 h (**B**). Cell viability was evaluated as described in Section 2.3. All values are reported as the mean ± SEM of six independent measurements. Statistical analysis was performed using one-way ANOVA with Tukey post hoc test. Significance was evaluated with respect to untreated cells (*: *p* < 0.05; **: *p* < 0.01).

The effect on HLE cell viability (evaluated through the MTT assay, see Section 2.3) induced upon exposure to different HNE concentrations, is reported in Figure 6. No effect was observed for a 6 h incubation in the presence of HNE concentrations up to 40 μM, while a reduction in cell viability of approximately 20% was observed in the presence of 50 μM of the aldehydic compound. A more pronounced effect was observed after 24 h of incubation. In this case, 40 and 50 μM HNE determined a reduction in cell viability of 26% and 27%, respectively. Comparable results were obtained when cell vitality was evaluated using crystal violet (Supplementary Materials, Figure S7).

**Figure 7:**
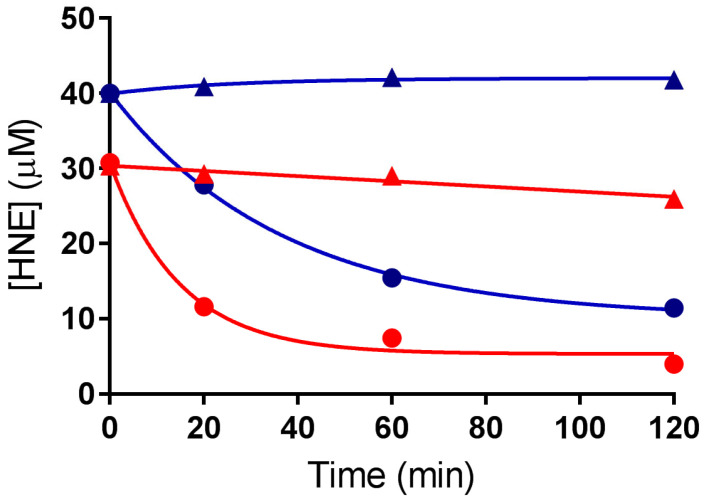
Removal of HNE from culture medium in the presence of HLE cells. HLE cells were grown as described in Section 2.2 and incubated in MEM in the presence of the following HNE concentrations: 30 μM, red symbols; 40 μM, blue symbols. Red and blue triangles refer to 30 μM and 40 μM HNE incubated in culture medium in the absence of HLE cells, respectively. HNE was evaluated as described in Section 2.9. Each value is the mean ± SD of three measurements, deriving from at least two independent experiments.

**Figure 8:**
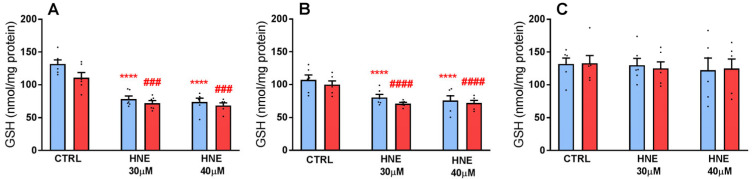
Effect of HNE incubation on intracellular glutathione content. GSH (blue bars) and total glutathione (red bars) levels were measured as described in Section 2.8, by HPCE analysis, in crude extracts obtained from HLE cells incubated for 30 min (**A**), 1 h (**B**), and 3 h (**C**), in the presence of the indicated HNE concentrations. Results are expressed as mean ± SEM of six independent experiments. Significance was evaluated with respect to GSH value (*) or total glutathione value (#) of control cells incubated in the absence of the aldehyde (CTRL bars), using one-way ANOVA followed by Tukey post hoc test (###: *p* <0.001, ****, ####: *p* <0.0001).

The ability of HLE cells to actively remove the aldehydic compound from the medium is clear from the data in Figure 7, in which a progressive decrease in HNE in the culture medium, comparable to what has been reported using ^3^H-labelled HNE [66], is observed. After 60 min of incubation, in the presence of 30 μM and 40 μM, approximately 25% and 33%, respectively, of the initial HNE concentration, were still measured in the medium. The removal of HNE from the medium was accompanied by changes in the intracellular glutathione levels. In fact, a significant, although transient, decrease in glutathione content of HLE cells was observed within 30–60 min (Figure 8A,B). No oxidation of glutathione occurred, since reduced and total glutathione levels decreased to the same extent with respect to levels measured in untreated cells. A complete recovery of glutathione content was observed after 180 min (Figure 8C), and maintained with further incubation, up to at least 6 h from the beginning of the incubation in the presence of HNE.

The generation of the adduct between GSH and HNE (i.e., GSHNE) is considered a relevant intracellular detoxification pathway of the aldehydic compound. Indeed, in HLE cells, it has been demonstrated that approximately 40% of the total HNE metabolism occurs through GSH conjugation [66]. Consistent with this observation, and with the decrease in intracellular glutathione content reported in Figure 8, is the fact that HPCE analysis of extracts from HLE cells treated with HNE, as above, revealed the generation of a molecular species, whose elution time was compatible with both GSHNE and its reduced form GSDHN.

**Figure 9:**
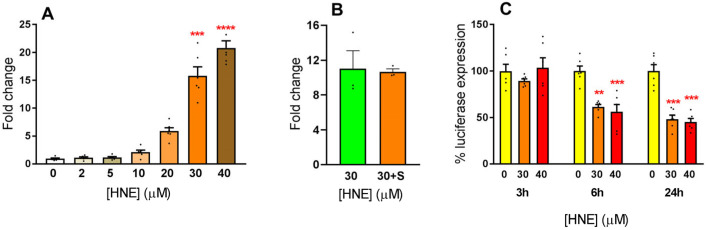
Inflammatory response to HNE treatment. (**A**) HLE cells were incubated in the presence of the indicated HNE concentrations and COX-2 expression was measured after 6 h, as described in Section 2.7. Values are the mean ± SEM of six independent measurements. Statistical analysis was performed through one-way ANOVA followed by Tukey post hoc test; significance was evaluated with respect to untreated cells (***: *p* ≤ 0.001 ****: *p* ≤ 0.0001) (**B**) HLE cells were incubated in the presence of the indicated HNE concentration, alone or in the presence of 50 μM sorbinil (S). Statistical analysis was performed using Student’s *t*-test. (**C**) NF-κB activation was evaluated after the indicated times of exposure to HNE and reported as % of luciferase expression measured for untreated cells. Values are the mean ± SEM of six independent measurements. Statistical analysis was performed through one-way ANOVA followed by Tukey post hoc test; significance was evaluated with respect of untreated cells at each time of exposure (**: *p* < 0.01; ***: *p* ≤ 0.001).

In our conditions, a marked increase in COX-2 expression resulted subsequent to HNE treatment with concentrations of at least 30 μM (Figure 9A). It is worth noting, that in these conditions, COX-2 expression was maximal at around 6–8 h from the beginning of the treatment; on the other hand, when HLE cells were exposed to high glucose levels, COX-2 expression was maximal after 48 h of treatment. Moreover, when HLE cells were exposed to HNE, in contrast to what was observed for the glucose-induced stress, but consistent with observations reported for other cell lines treated with HNE [67,68], COX-2 expression appeared to not be linked to NF-κB. In fact, no activation of NF-κB was observed (Figure 9C), at least from 3 to 24 h from the beginning of the HNE treatment. Indeed, HNE appeared to significantly decrease NF-κB activation, with respect to basal levels. This effect is consistent with what was observed for hepatocytes exposed to similar HNE concentrations [69]. Moreover, as observed when high glucose was used to induce an inflammatory response, also in the case of HNE, COX-2 expression was not affected by the presence of sorbinil (Figure 9B).

**Figure 10:**
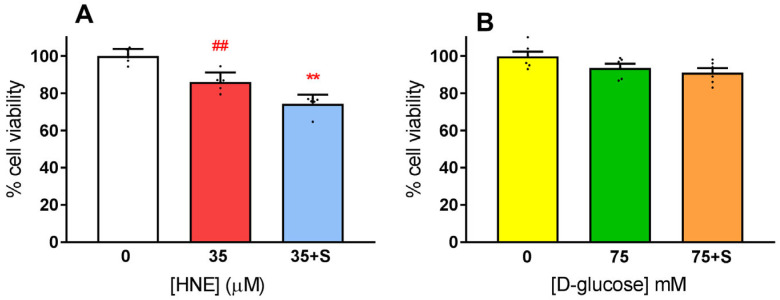
Effect of AKR1B1 inhibition on cell viability of HLE cells undergoing different stress. HLE cells were grown as described in Section 2.2; cell viability was evaluated as described in Section 2.3, and expressed as % with respect to the value measured for untreated cells. (**A**) HLE cells were maintained for 6 h in MEM containing 0.05% DMSO, in the presence of the indicated HNE concentration. S refers to the addition of 10 µM sorbinil. Values are the mean ± SEM of six independent measurements. Statistical analysis was performed using one-way ANOVA with Tukey post hoc test. Significance was evaluated with respect to untreated cells (^##^: *p* < 0.01), or with respect to cells incubated with 35 µM HNE (**: *p* < 0.01). (**B**) HLE cells were maintained for 24 h in MEM containing 0.05% DMSO, in the presence of the indicated D-glucose concentration. S refers to the addition of 10 µM sorbinil. Values are the mean ± SEM of six independent measurements. Statistical analysis was performed using one-way ANOVA with Tukey post hoc test. Significance was evaluated with respect to untreated cells.

The absence of NF-κB activation reported here, and also observed, as stated above, in other cells lines exposed to HNE [67,68], raises a question about the extent of involvement of GSHNE, upon its reduction to GSDHN, in NF-κB activation [43]. In fact, the absence of NF-κB activation is discordant with the reported occurrence of GSHNE as a relevant metabolite in HLE cells exposed to HNE [66]. In any case, when considering the possible route of HNE transformation, besides the adduct formation, previous data [65,66] indicated the occurrence of reductive phenomena directly on the aldehydic compound. These reactions have been partly ascribed to the NADPH-dependent action of AKR1B1. Indeed, a relevant active role of AKR1B1, in HLE cells exposed to HNE treatment, came from results of Figure 10, in which the effect of sorbinil on cell viability in the presence of HNE is reported. It is evident how, in this case, AKR1B1 inhibition exacerbated the toxic effect exerted by HNE on cell viability, as observed for the cytotoxicity induced by some alkanals [70]. Figure 10 also shows that the same sorbinil concentration did not affect cell viability using the exposure to 75 mM D-glucose as the stress condition. Thus, due to this detoxifying action of AKR1B1, it is difficult to predict and rationalize what would be the effect of enzyme inhibition on the inflammatory response. In any case, the view of AKR1B1 inhibition as an easy tool to generally counteract all inflammatory events, is somewhat simplistic.

## 4. Conclusions

This work highlighted the damaging events occurring upon exposure of an HLE cell line to hyperglycemic or oxidative stress conditions. Both conditions resulted in the onset of a marked inflammatory response, in terms of increased COX-2 expression, which, only in the case of hyperglycemic stress, was mediated by NF-κB activation. The observation that, in our cell model, the inflammation consequent to high glucose exposure was not minimally impaired by completely blocking the polyol pathway flux through AKR1B1 inhibition, suggested the contribution to inflammation of other (probably very early) alterations, which, at least in our conditions, cannot be counteracted by blocking the AKR1B1 activity. This enzyme was undoubtedly confirmed as the only factor responsible for the increased polyol pathway flux occurring in hyperglycemic conditions, and AKR1B1 pharmacological inhibition still remains a tool to fight diabetic complications strongly linked to sorbitol accumulation. On the other hand, a clear assessment of factors contributing to the onset of the NF-κB-mediated COX-2 expression is still needed, in order to identify possible additional enzymatic targets, whose impairment may prove useful in attenuating the onset of the high-glucose-induced inflammatory response. Concerning the HNE-induced stress, our data strongly supports the protective role of AKR1B1, which contributes to HNE detoxification. These data confirm the view of AKR1B1 as having complex activity, able to act simultaneously both as a damaging and protecting factor, thus reinforcing the relevance of the so-called AKR1B1 “differential inhibition” approach [16], aimed at blocking the enzyme in its glucose-dependent activity, leaving its detoxifying function unaffected.

## Data Availability

The data presented in this study are available in the article and Supplementary Materials; upon request, the raw data will be made available by the corresponding author.

## Supplementary Materials

The following supporting information can be downloaded at: https://www.mdpi.com/article/10.3390/antiox12040829/s1, Purification of human recombinant sorbitol dehydrogenase; Figure S1. Elution profile of the DEAE-Sepharose CL-6B chromatography column; Figure S2. Elution profile of the Affi-Blue gel chromatography column; Figure S3. SDS-PAGE analysis; Figure S4. Effect of sorbinil on cell viability; Figure S5. Effect of sorbinil on sorbitol accumulation; Figure S6. Effect of sorbinil on NF-κB activation; Figure S7. Effect of HNE on cell vitality.
