# Supplementary material for: Response of a Human Lens Epithelial Cell Line to Hyperglycemic and Oxidative Stress: The Role of Aldose Reductase
Source: Antioxidants (Basel). 2023 Mar 28;12(4):829. doi: 10.3390/antiox12040829 (PMC10135174; doi:10.3390/antiox12040829)
Supplement: Supplementary file 1 [file antioxidants-12-00829-s001.zip › antioxidants-2253756-supplementary.pdf]

Response of a Human Lens Epithelial Cell Line to Hyperglycemic and Oxidative Stress: the Role of Aldose Reductase

Gemma Sardelli, Viola Scali, Giovanni Signore, Francesco Balestri, Mario Cappiello, Umberto Mura, Antonella Del Corso and Roberta Moschini

SUPPLEMENTARY MATERIALS

Purification of human recombinant sorbitol dehydrogenase.

During the purification steps the sorbitol dehydrogenase (SDH) activity was assayed at 37°C using D-fructose as substrate, measuring the decrease in absorbance at 340 nm, which corresponds to NADH oxidation. The reaction mixture (0.7 ml final volume) contained 0.24 mM NADH and 0.4 M D-fructose in 100 mM Tris-HCl buffer, pH 7.4; the reaction was initiated by addition of the substrate. One unit of enzyme activity is defined as the amount of SDH that catalyzes the oxidation of 1 μmol of NADH/min. Human SDH was expressed in *Escherichia coli* BL21 cells containing an expression plasmid pET-30a-hSDH (Eurofins) and purified using an anion-exchange chromatography and a dye-affinity chromatography. Briefly the crude extract was applied to a DEAE-Sepharose CL-6B (Millipore) column (2.8 x 8.5 cm) equilibrated with 50 mM sodium phosphate buffer pH 7.0 (P buffer) containing 0.5 mM DTT. The flow rate was 20 mL/h, and 3-mL fractions were collected. Fractions showing SDH activity (Figure S1) were pooled, concentrated using an Amicon YM30 ultrafiltration membrane and applied on an Affi-Gel Blue (Bio Rad) column (1.4 x 7 cm) previously equilibrated with P buffer. The flow rate was 25 mL/h and 2.5 mL fractions were collected. The column was initially eluted with P buffer until the absorbance at 280 nm was approximately 0.3 and then with the same buffer supplemented with 0.37 M NaCl until the absorbance at 280 nm of the fractions reached the basal value. Finally, SDH was eluted with P buffer supplemented with 0.37 M NaCl, 0.1 mM NAD^+ and 2 mM DTT (Figure S2). The homogeneity of the protein preparation was verified by SDS-PAGE (Figure S3). The specific activity of the purified enzyme (measured in the above conditions) was 269 ± 15 U/mg. The purified enzyme was stored at -80°C in a 20% (v/v) glycerol solution until used.

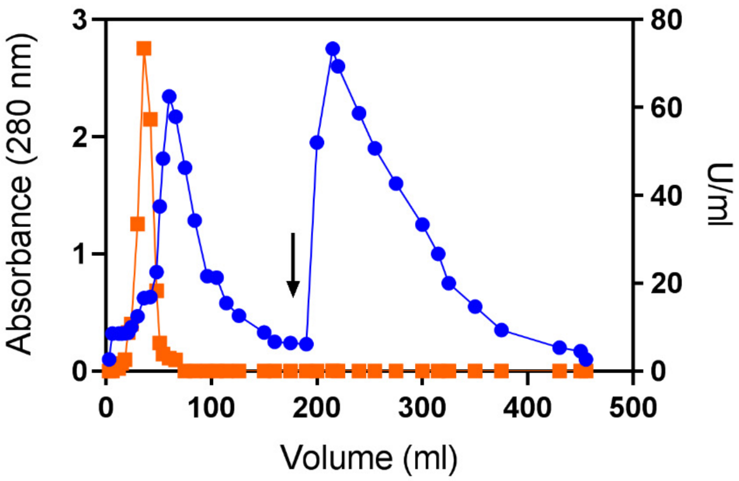

Figure S1. Elution profile of the DEAE-Sepharose CL-6B chromatography column. Blue and orange symbols refer to absorbance at 280 nm and sorbitol dehydrogenase activity, respectively. The arrow refers to the addition of 0.5 M NaCl to the elution buffer.

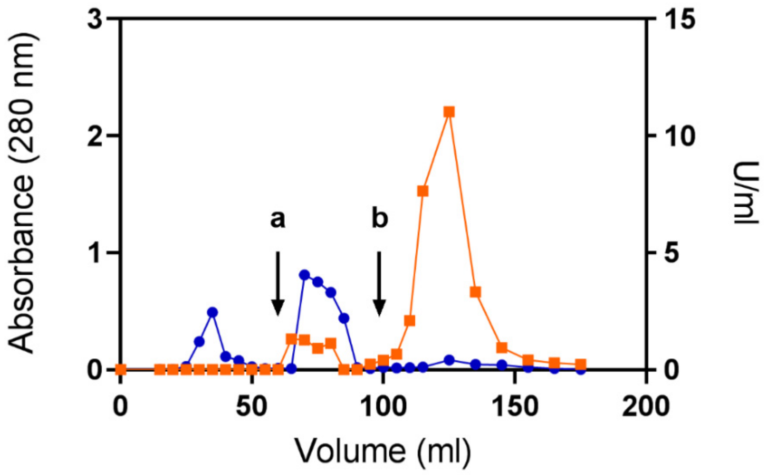

Figure S2. Elution profile of the Affi-Blue gel chromatography column. Blue and orange lines refer to absorbance at 280 nm and sorbitol dehydrogenase activity, respectively. The arrows a and b refer to the addition to the elution buffer of 0.37 M NaCl alone or supplemented with 0.2 mM NAD^+ and 2 mM DTT, respectively.

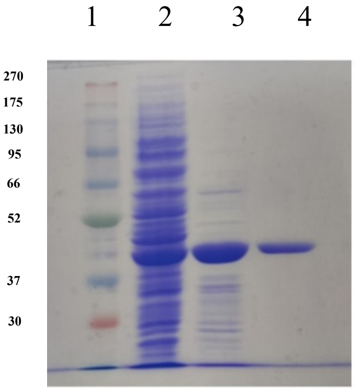

Lane 1: Molecular weight standard

Lane 2: 25 µg Crude extract

Lane 3: 10 µg Pool DEAE Sepharose CL-6B

Lane 4: 1 µg Pool Affi-Gel Blue

Figure S3. SDS-PAGE analysis. Samples from different steps of the purification process were subjected to SDS-PAGE, using a 12% (w/v) acrylamide gel, followed by staining with Coomassie brilliant blue. Numbers alongside refer to molecular mass of protein standards.

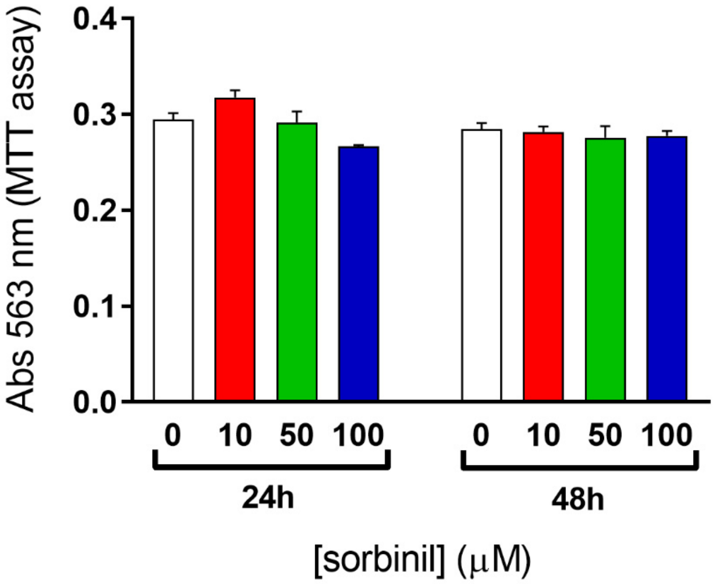

Figure S4. Effect of Sorbinil on cell viability. HLE cells were grown as described in *Section 2.2* and maintained for the indicated times in MEM supplemented with 0.05% DMSO in the presence of the indicated Sorbinil concentrations. Cell viability was evaluated as described in *Section 2.3*. Values are reported as the mean ± SEM of six independent measurements. Statistical analysis was performed using one way ANOVA with Tukey post hoc test. Significance was evaluated with respect to white bars.

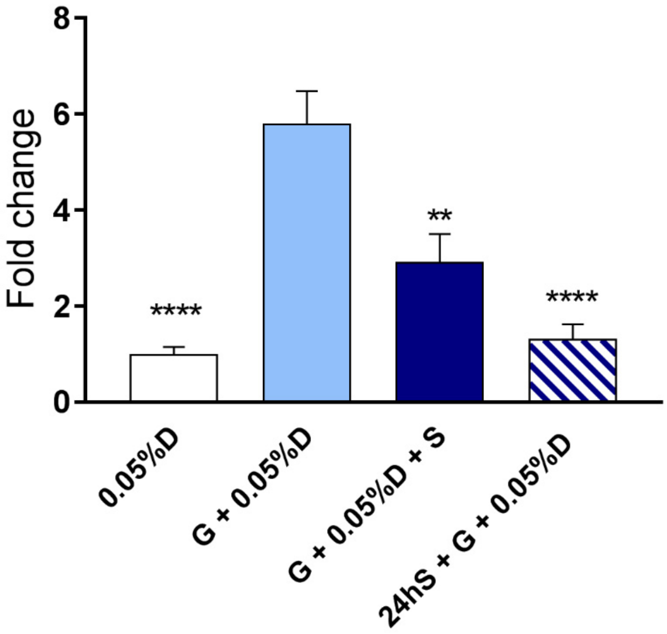

Figure S5. Effect of Sorbinil on sorbitol accumulation. HLE cells were grown for 24 h in MEM containing 0.05 % DMSO (D) alone or supplemented with: 75 mM D-glucose (G); 10 μM Sorbinil (S). 24h S refer to a 24 h treatment with 10 μM Sorbinil before the exposure to 75 mM D-glucose. Sorbitol content is expressed as fold change with respect to values measured in MEM. All values are reported as the mean ± SEM of six independent measurements. Statistical analysis was performed using one way ANOVA with Tukey post hoc test. Significance was evaluated with respect to cells incubated in 75 mM D-glucose (**: $p < 0.01$; ****: $p < 0.0001$).

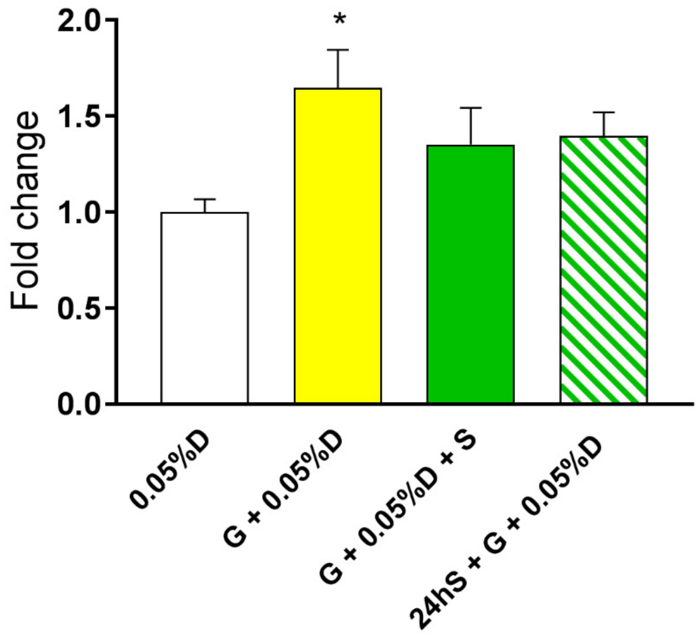

Figure S6. Effect of Sorbinil on the inflammatory response of HLE cells exposed to hyperglycemic conditions. NF-kB activation was evaluated in HLE cells grown in MEM containing 0.05 % DMSO (D) supplemented with: 75 mM D-glucose (G); 100 μM Sorbinil. (S). 24h S refer to a 24 h treatment with 100 μM Sorbinil before the exposure to 75 mM D-glucose. Data are expressed as fold change with respect to values measured in MEM alone. All values are reported as the mean ± SEM of six independent measurements. Statistical analysis was performed using one way ANOVA with Tukey post hoc test. Significance was evaluated with respect to cells incubated in MEM containing 0.05 % DMSO (*: $p < 0.05$).

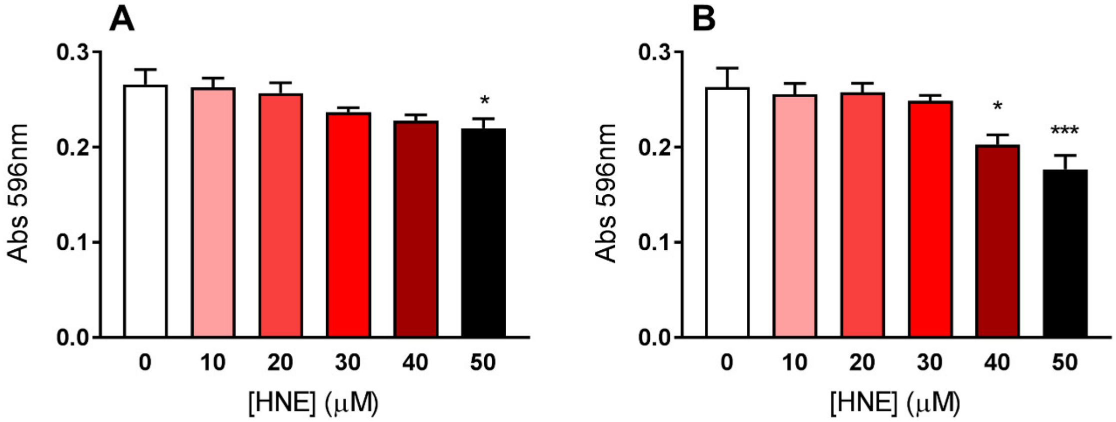

Figure S7. Effect of HNE on cell vitality. HLE cells were grown as described in Section 2.2 in MEM in the presence of the indicated HNE concentrations for 6 (A) and 24 h (B) and cell vitality was measured using crystal violet (see Section 2.3). Values are reported as the mean ± SEM of six independent measurements. Statistical analysis was performed using one way ANOVA with Tukey post hoc test. Significance was evaluated with respect to untreated cells.
